# Risk of influenza and COVID-19 illness and pediatric obstructive sleep apnea: a TriNetX cohort with 5-year follow-up

**DOI:** 10.1007/s44470-025-00035-x

**Published:** 2026-02-11

**Authors:** Alex Gileles-Hillel, Joel Reiter, David Gozal

**Affiliations:** 1 Faculty of Medicine, The Hebrew University, Jerusalem, Israel; 2 Pediatric Pulmonology and Sleep Unit, Pediatric Division, Hadassah Medical Center, Jerusalem, Israel; 3 Office of The Dean and Departments of Pediatrics and Biomedical Sciences, Joan C. Edwards School of Medicine, Marshall University, Huntington, WV USA

## Abstract

**Background:**

Obstructive sleep apnea (OSA) is associated with impaired immune responses. Prior small-scale investigations suggested that pediatric OSA leads to a more severe clinical course of common childhood viruses. Whether OSA increases the risk of infection remains unknown.

**Objectives:**

To examine whether children with OSA are at increased risk of influenza or COVID-19, and to assess the effect of adenotonsillectomy.

**Methods:**

Using TriNetX, we compared children aged 2–18 years with incident OSA and matched controls without OSA. Outcomes were ICD-10–coded influenza and COVID-19 over a five-year follow-up. We estimated risk ratios (RRs) and Kaplan–Meier hazard ratios (HRs).

**Results:**

Matched cohorts included 539,127 children each (mean age 5.6 ± 3.6 years). Influenza was diagnosed in 5.1% of OSA vs. 2.8% of controls (RR 1.80; 95% CI, 1.765–1.836); five-year influenza-free survival was 90.27% vs. 93.04% (HR 1.45; 95% CI, 1.421–1.479). COVID-19 was diagnosed in 2.5% vs. 1.0% (RR 2.496; 95% CI, 2.418–2.576); five-year COVID-19-free survival was 95.02% vs. 97.49% (HR 1.986; 95% CI, 1.924–2.050). Effects were similar across age groups. In the treatment sub-analysis (*n* = 96,004 per group), adenotonsillectomy did not reduce risk. In secondary analyses, OSA was also associated with a higher risk of pneumonia due to influenza or COVID-19.

**Conclusions:**

In a period spanning 5 years after OSA diagnosis, children of all ages have a significantly higher risk of influenza and COVID-19 diagnoses. Although absolute risks are low, adenotonsillectomy does not lessen susceptibility, suggesting persistent immune dysregulation and supporting prioritization of seasonal vaccination in children with OSA.

**Supplementary Information:**

The online version contains supplementary material available at 10.1007/s44470-025-00035-x.

## Brief summary

**Current knowledge/study rationale:**

The biologic link between pediatric OSA and respiratory infections is plausible, but pediatric data on influenza and COVID-19 risk are limited. We used the TriNetX database to analyze a cohort of children and adolescents newly diagnosed with OSA over 5 years, including COVID-19 as a comparator.

**Study impact:**

Children with OSA were about twice as likely to be diagnosed with influenza or COVID-19 as matched controls across age groups. Sub-analysis by treatment suggested that adenotonsillectomy did not reduce this increased risk, underscoring the importance of seasonal vaccination in children with OSA and motivating work on mechanisms and alternative management.

**Supplementary Information:**

The online version contains supplementary material available at 10.1007/s44470-025-00035-x.

## Introduction

Obstructive sleep apnea (OSA) affects an estimated 1–4% of otherwise healthy children and is associated with somatic growth impairments, behavioral and neurocognitive morbidities, and cardiometabolic risk [1]. Seasonal influenza has been and remains a leading cause of acute respiratory illness in childhood, with clear year-to-year variability in circulation patterns, strain virulence, and test positivity that collectively reflect changes in viral epidemiology, clinical practice, and public health measures [2]. More recently, the emergence of the SARS-CoV-2 pandemic has contributed to the sustained intercurrent respiratory illness burden in the pediatric age group [3, 4]. The biologic and clinical intersection between pediatric sleep-disordered breathing (SDB) and respiratory viral infections is epidemiologically and biologically plausible and increasingly recognized, yet data focused specifically on influenza and COVID-19 in children with OSA are limited [5]. 

Evidence from various contexts supports a link between OSA and heightened susceptibility to infection in childhood. First, upper airway lymphoid hypertrophy and recurrent inflammation and infections are the main pathophysiological characteristics of pediatric OSA, conditions that intrinsically perturb local airway immunity and mucociliary defenses [6, 7]. Second, peripheral immune profiling suggests altered cellular immunity in pediatric OSA, including changes in B cells, CD8⁺ T cells and CD4/CD8 balance, potentially compromising host antiviral responses [8–10]. Furthermore, and irrespective of a formal OSA diagnosis, insufficient and poor quality sleep increase the risk of upper respiratory tract infections, highlighting sleep as a modifiable determinant of viral susceptibility [11, 12]. Together, these findings raise the possibility that children with OSA may be more likely to contract influenza or COVID-19, and/or follow a more complicated clinical course once infected.

Population-level studies in adults and mixed-age cohorts have begun to quantify this risk. In a nationwide analysis, adult OSA was associated with increased odds of influenza-associated severe acute respiratory infection [13]. Pediatric-specific outcome data, while sparse, suggest a similar signal: during influenza hospitalizations, children with OSA had longer length of stay, greater hospital costs, and higher odds of pneumonia compared with matched peers without OSA [14]. Furthermore, the reverse relationship has also been identified, namely, frequent respiratory viral illnesses favoring the occurrence of OSA in childhood [15–18]. Nonetheless, there are no large-sample epidemiological studies that address the question as to whether OSA constitutes an independent risk factor for contracting influenza or COVID-19 in children.

In the COVID-19 era, SARS-CoV-2 testing became a routine component of multiplex respiratory testing in children and provides a relevant comparator pathogen when examining test positivity patterns. Emerging literature in adults and mixed-age cohorts suggests that pre-existing OSA may increase susceptibility to COVID-19, worsen acute outcomes, and elevate the risk of post-acute sequelae; pediatric data are more limited but hint toward adverse outcome rates among children with OSA who are diagnosed with COVID-19 [19, 20]. Including COVID-19 as a parallel outcome can therefore contextualize any OSA-influenza associations within broader viral testing practices and immunopathologic risk.

To address these gaps, we leveraged TriNetX—a federated real-world data network that provides de-identified, standardized electronic health record (EHR) data and analytic tools across large, multi-institution pediatric cohorts—to examine whether children with OSA are more likely to get diagnosed with influenza compared with matched children without OSA. Specifically, we used the TriNetX Pediatric Collaboratory Network to assemble a cohort of children with an index diagnosis of OSA and prospectively evaluated diagnoses of influenza over a five-year follow-up window, while also assessing diagnosis of COVID-19 as a comparator outcome. We hypothesized that OSA would be associated with a higher diagnosis of these two viral infections.

## Methods

We conducted a retrospective cohort study on the TriNetX Global Collaborative Network using the Compare Outcomes workspace (data extracted on September 9, 2025). TriNetX aggregates de-identified electronic health record data from participating healthcare organizations in a federated environment.

### Cohorts and index

Children aged 2 to 18 years were assigned to the OSA cohort (ICD-10-CM G47.33 or G47.3 recorded at ≤ 18 years) or to a Control cohort (no G47.33/G47.3 at ≤ 18 years). The index date was the first qualifying OSA code (OSA) or an ambulatory visit (Control). Follow-up began 1 day after the index and continued for 1,825 days. None of the controls were diagnosed with OSA during the follow-up.

### Outcomes and analyses

Primary outcomes were influenza (ICD-10-CM J10/J11) and COVID-19 (U07.1). Risk and Kaplan–Meier (KM) analyses excluded prior outcome. Propensity score matching (PSM) was 1:1 on Age at Index and overweight and obesity (E65–E68). Post-match sample sizes were *n* = 539,127 per cohort.

### Prespecified age strata

To evaluate whether specific age-dependent immunologic milestones underlie age-dependent clustering of cases [21, 22], we repeated the matched analyses within three age bands: 2 to 6 years (*n* = 100,652 per cohort), 6 to 12 years (*n* = 265,980 per cohort), and 12 to 18 years (*n* = 232,944 per cohort). All other settings paralleled the primary analysis.

### Treatment subanalysis

For the treatment comparisons, we defined adenotonsillectomy using the following procedure codes in TriNetX: CPT 42,830 (adenoidectomy, primary; younger than age 12), CPT 42,831 (adenoidectomy, primary; age 12 or over), CPT 1,007,178 (tonsillectomy and adenoidectomy), CPT 1,007,184 (adenoidectomy, primary), and SNOMED 119,954,001 (adenoid excision). Children in the treated cohort were required to have an OSA diagnosis (ICD-10-CM G47.33 and G47.3) recorded at least 1 day before any qualifying treatment code; the untreated cohort required OSA but excluded any of the treatment codes. We set the index date as the first OSA diagnosis for the untreated cohort and the first qualifying surgery after OSA for the treated cohort; outcomes were assessed from 1 day to 1,825 days after each cohort’s index. Propensity score matching was performed as above. After matching, both the treated and untreated groups comprised 96,004 children each.

## Results

After matching, each of the two cohorts (i.e., OSA and Control) included 539,127 children (mean age 5.6 ± 3.6 years). Sex and race distributions are shown in Table 1.

**Table 1:** Cohort characteristics after propensity score matching (PSM)

Characteristic	OSA (*n* = 539,127)	Control (*n* = 539,127)
Age at index, years (mean ± SD)	5.6 ± 3.6	5.6 ± 3.6
Male, n (%)	304,835 (56.5)	284,201 (52.7)
White race, n (%)	292,795 (54.3)	241,432 (44.8)

PSM variables: Age at Index; Overweight, obesity and other hyperalimentation (E65–E68). Counts and percentages are directly from TriNetX

*ICD-10-CM* International Classification of Diseases, Tenth Revision, Clinical Modification; *n* number; *OSA* obstructive sleep apnea; *PSM* propensity score matching; *SD* standard deviation

### Influenza

Among children at risk (508,107 OSA and 532,045 Control), 25,814 and 15,018 were diagnosed with influenza illness across the 5-year follow-up period. The risk ratio for the OSA cohort was 1.800 (95% CI, 1.765 to 1.836). In addition, the 5-year influenza-free survival was 90.27% in OSA versus 93.04% in Control (HR 1.450; 95% CI, 1.421 to 1.479; log rank χ2 1325.308, *P* <.001) (Table 2).

**Table 2:** Influenza outcomes over 5-year follow-up

Analysis	Metric	OSA	Control
Risk (excluding prior outcome*)	Patients in cohort	508,107	532,045
	Patients with outcome	25,814	15,018
	Risk	0.051	0.028
	Risk difference (95% CI)	—	0.023 (0.022–0.023)
	Risk ratio (95% CI)	—	1.800 (1.765–1.836)
KM survival (excluding prior outcome*)	Survival at 5 years	90.27%	93.04%
	Log-rank χ2 (df, P)	—	1325.308 (1, < 0.001)
	Hazard ratio (95% CI)	—	1.450 (1.421–1.479)

* Exclusions for risk/survival analyses: 31,020 OSA and 7,082 controls had influenza prior to the time window and were excluded

*CI* confidence interval; *df* degrees of freedom; *HR* hazard ratio; *KM* Kaplan–Meier; *n* number; *OSA* obstructive sleep apnea

### COVID-19

In matched cohorts (*n* = 539,127 each), 13,134 OSA and 5,372 Control patients had COVID-19 recorded in risk analyses that excluded prior outcome. The risk ratio among OSA affected children was 2.496 (95% CI, 2.418 to 2.576). In KM analyses (excluding prior outcome), the 5-year survival was 95.02% in OSA versus 97.49% in Control (HR 1.986; 95% CI, 1.924 to 2.050; log rank χ2 1866.747, *P* <.001) (Table 3).

**Table 3:** COVID-19 outcomes over 5-year follow-up

Analysis	Metric	OSA	Control
Risk (excluding prior outcome*)	Patients in cohort	525,059	535,990
	Patients with outcome	13,134	5,372
	Risk	0.025	0.010
	Risk difference (95% CI)	—	0.015 (0.014–0.015)
	Risk ratio (95% CI)	—	2.496 (2.418–2.576)
KM survival (excluding prior outcome*)	Patients at risk	525,059	535,990
	Events	13,134	5,372
	Survival at 5 years	95.02%	97.49%
	Log-rank χ2 (df, P)	—	1866.747 (1, < 0.001)
	Hazard ratio (95% CI)	—	1.986 (1.924–2.050)

*Exclusions for survival analyses: 14,068 OSA and 3,137 controls had COVID-19 prior to the time window and were excluded

*CI* confidence interval; *COVID-19* coronavirus disease 2019; *df* degrees of freedom; *HR* hazard ratio; *KM* Kaplan–Meier; *n* number; *OSA* obstructive sleep apnea

### Age strata

Findings were consistent across the 3 developmental age groups. For influenza illness, risk ratios ranged from 1.79 to 1.89, and hazard ratios 1.27 to 1.32. For COVID-19, risk ratios ranged from 2.36 to 2.52 and hazard ratios were 1.62 to 1.82. Complete age-stratified outputs are provided in the Online Supplemental Results.

### Treatment subanalysis (adenotonsillectomy vs. no surgery)

After 1:1 propensity matching, the treated and untreated OSA cohorts each included 96,004 children. For influenza, untreated versus treated risks across 5 years were 4.9% (4,455/90,903) and 5.6% (5,005/88,638), respectively (*p* <.001), yielding a risk ratio of 0.868 (95% CI, 0.834 to 0.903). Five-year influenza-free survival was 90.89% in untreated and 89.67% in treated children; HR 0.879 (95% CI, 0.845 to 0.916, *P* =.448); log-rank χ2 38.959, *P* <.001. For COVID-19, untreated versus treated risks were 2.5% (2,332/93,626) and 2.6% (2,380/92,716), respectively (*P* =.173), risk ratio 0.970 (95% CI, 0.917 to 1.027); five-year survival was 95.18% and 94.97%, respectively; HR 0.993 (95% CI, 0.938 to 1.051); log-rank χ2 0.064, *P* =.800.

### Additional sensitivity analyses (era, co-morbidities, severity)

To account for changes imposed by the COVID-19 pandemic on healthcare testing practices, we examined the influenza risk only in those who presented before 1.1.2015, making the 5-year follow-up period entirely prior to the COVID-19 the pandemic. Among children at risk (121,705 OSA and 121,705 Control), 7,902 and 3,642 were diagnosed respectively with clinical influenza illness across the 5-year follow-up period. The risk ratio for the OSA cohort was 2.172 (95% CI, 2.09 to 2.257). In addition, the 5-year influenza-free survival was 93.04% in OSA versus 95.61% in Control (HR 1.606; 95% CI, 1.544 to 1.67; log rank χ2 569.193, *P* <.001).

Since some pediatric cohort subsets may experience both increased risk of OSA and of viral infections, we also performed the propensity score matched analysis after excluding children who are at increased risk of both OSA and viral infection due to underlying comorbidity (e.g., Trisomy 21, Asthma, Prader-Willi). Among matched remaining cohorts (*n* = 380,443 each), 13,051 and 9,633 were diagnosed with influenza illness across the 5-year follow-up period in OSA and controls respectively. The risk ratio for the OSA cohort was 1.401 (95% CI, 1.365 to 1.438). In addition, the 5-year influenza-free survival was 92.26% in OSA versus 93.174% in Control (HR 1.164; 95% CI, 1.134 to 1.195; log rank χ2 128.034, *P* <.001). For COVID-19, 6,841 and 3,488 were diagnosed with COVID-19 illness across the 5-year follow-up period in OSA and controls respectively, with risk ratio of 1.996 (95% CI, 1.917 to 2.078). The 5-year COVID-19-free survival was 95.89% in OSA versus 97.56% in Control (HR 1. 649; 95% CI, 1.583 to 1.718; log rank χ2 590.068, *P* <.001).

Finally, to examine how OSA influences the risk for severe viral infection, we examined specifically cases of pneumonia due to influenza (ICD-10 codes J10.0, J11.0, J09.X1) and COVID-19 (J12.82) in all the children with OSA and their matched controls (*n* = 506,177 in each group). For influenza pneumonia, 866 events occurred in the OSA cohort and 322 in controls over the 5-year follow-up, yielding risks of approximately 0.2% and 0.1%, respectively, and a risk ratio of 2.69 (95% CI, 2.37–3.06); the 5-year influenza pneumonia–free survival was 99.68% in OSA versus 99.85% in control (HR 2.10; 95% CI, 1.84–2.38; log-rank χ² 134.5; *P* <.001). For COVID-19 pneumonia, 467 children with OSA and 18 controls were affected, corresponding to risks of approximately 0.1% and < 0.01% and a risk ratio of 25.96 (95% CI, 16.21–41.57); the 5-year COVID-19 pneumonia–free survival was 99.82% in OSA versus 99.99% in control (HR 20.16; 95% CI, 12.59–32.29; log-rank χ² 315.6; *P* <.001).

Taken together, these sensitivity analyses further strengthen the evidence for an increased risk of influenza and COVID-19 clinically symptomatic infection and pneumonia in otherwise healthy children with OSA.

## Discussion

In the present study spanning a 5-year follow-up period, we found that children and adolescents who were newly diagnosed with OSA were two-fold more likely to be diagnosed with influenza or with COVID-19-related illness, even after adjusting for age and obesity status. Despite differences in viral positivity between age groups, the effect of OSA on clinical illness risk was similar in magnitude across strata. Similarly, although certain comorbidities are known to increase the risk of both OSA and viral infection, the association between OSA and infection risk remained evident among children without comorbidities and, for influenza, in the pre-pandemic era. When we restricted the outcome to pneumonia secondary to influenza or COVID-19 as a proxy for severe viral illness, OSA remained associated with a markedly increased risk. These patterns were evident in both cumulative risk and time-to-event analyses. Treatment of OSA did not alter this risk significantly.

Before discussing the implications of our findings, several methodological considerations deserve commentary. First, because the study design is retrospective in nature, direct causality cannot be inferred. Notwithstanding, we focused on newly diagnosed OSA and excluded children with prior influenza or COVID-19 illness. Thus, tracking children prospectively for 5 years after the index date provides temporal support for an association between OSA and subsequent viral infection and related illness. Second, we did not have access to the vaccination records and, therefore, could not adjust for or stratify by influenza or COVID-19 vaccination status. Third, patients with a diagnosis of OSA may be more likely to seek medical care for respiratory symptoms and, therefore, be more likely to be diagnosed with influenza/COVID-19. Finally, the absolute risk for each of the viral illnesses was low in our cohort, compared to seasonal patterns in the pediatric population. It is plausible that the data presented here reflects the more severe cases seeking medical care and, therefore, getting diagnosed and coded. This interpretation is supported by the sub-analysis of cases diagnosed with pneumonia.

The increased risk of influenza or COVID-19 illness among children with OSA has several important implications for children and pediatric health providers. It is now well established that OSA is associated with multiple morbidities, including cardiovascular and neurocognitive and behavioral consequences [23, 24]. The increased risk for viral infection further underscores the importance of timely diagnosis of sleep-disordered breathing. In the era of vaccine hesitancy and parental concern about risks versus benefits of seasonal influenza vaccination, our findings should reinforce clinicians in their quest to encourage families of children with OSA to pursue vaccination. Given the 2-fold elevation in risk, OSA could be viewed as a practical flag for preventive care. Similar to other respiratory disorders, at the time of OSA diagnosis, clinicians may wish to prioritize seasonal influenza vaccination and ensure that COVID-19 immunization is up to date. Framing OSA as a “risk marker” may help overcome hesitancy during counseling. This is further supported by our current and previously published data suggesting not only a higher risk of infection but also worse outcomes in children with OSA infected with influenza or COVID-19 [14, 20].

The dysregulation of innate and adaptive immune milieus in pediatric OSA may explain both the increased susceptibility to viral infection and the more severe symptoms that prompt medical attention and diagnostic coding. The important question is the role of OSA treatment in mitigating this susceptibility. Our analysis suggests that adenotonsillectomy may not reduce this increased risk. This *prima facie* disappointing result of such exploratory analysis may reflect two clinical and pathophysiological observations. First, a significant proportion of children with OSA have residual sleep apnea after adenotonsillectomy, with rates ranging from 30 to 50% across cohorts [23, 25–27]. Second, OSA-related immune dysregulation could impair recruitment of an appropriate immune response, potentially reducing vaccine effectiveness against infection. OSA may induce immune dysregulation in part through methylation changes in immune cells [9, 28], and these changes are relatively stable and not easily reversed. Considering the previously observed reduced vaccine efficacy in patients with disrupted sleep, as occurs in OSA [29, 30], current findings underscore the added importance of vaccinating children diagnosed with OSA against these common viruses and, potentially, open the way to exploring improved vaccination protocols for this higher-susceptibility group.

A final notion relates to the increased COVID-19 risk in this cohort as compared to influenza. While a true increase in susceptibility as compared with influenza is plausible, this study spans the pandemic era, when testing was widespread and often performed even in the absence of symptoms, in contrast to the typical practice for influenza. There was marked variability and evolution in medical care practices from the pre-pandemic era to the present. Differences in testing intensity may therefore have contributed to the higher observed COVID-19 risk. Nonetheless, we examined the influenza risk in the pre-pandemic era and found an identical magnitude of the effect, suggesting that testing practices do not seem to account for the increased risk observed herein.

In summary, we found that in the 5 years following a diagnosis of OSA, children of any age are at significantly increased risk of being diagnosed with influenza and COVID-19 illness and of suffering from pneumonia due to these pathogens. While the absolute risk is relatively low, this increased susceptibility is not reduced by adenotonsillectomy alone and may reflect persistent immune dysregulation in this population.

## Supplementary Information

Below is the link to the electronic supplementary material.

Supplementary Material 1

## Data Availability

The datasets used and/or analyzed during the current study are available from the corresponding author on reasonable request.

## Abbreviations

ICD-10-CM: International Classification of Diseases, Tenth Revision, Clinical Modification
n: number
OSA: obstructive sleep apnea
PSM: propensity score matching
SD: standard deviation
