# Supplementary material for: Risk of influenza and COVID-19 illness and pediatric obstructive sleep apnea: a TriNetX cohort with 5-year follow-up
Source: J Clin Sleep Med. 2026 Feb 11;22(1):31. doi: 10.1007/s44470-025-00035-x (PMC12994981; doi:10.1007/s44470-025-00035-x)
Supplement: Supplementary file 1 — Supplementary Material 1 [file 44470_2025_35_MOESM1_ESM.docx]

**SUPPLEMENTAL RESULTS**

Age 2 to 6 years (post-match n = 100,652 per cohort)

Influenza. Across 5 years, 4,376 of 95,747 children with OSA (4.6%) versus 2,395 of 99,026 controls (2.4%) were diagnosed with influenza; risk ratio 1.89 (95% CI, 1.80 to 1.99). KM analysis (excluding prior influenza) showed five-year influenza-free survival 81.24% versus 82.06%; HR 1.27 (95% CI, 1.21 to 1.33); log-rank χ2 87.615, P < .001.

COVID-19. Risk analysis (including prior outcome) was 2.8% versus 1.1%; risk ratio 2.45 (95% CI, 2.29 to 2.63). In KM analyses (excluding prior outcome), five-year survival was 92.79% versus 94.99%; HR 1.62 (95% CI, 1.51 to 1.74); log-rank χ2 172.439, P < .001.

Age 6 to 12 years (post-match n = 265,980 per cohort)

Influenza. Across 5 years, 13,809 of 247,959 (5.6%) with OSA versus 8,037 of 261,737 (3.1%) controls had influenza; risk ratio 1.81 (95% CI, 1.77 to 1.86). KM showed five-year survival 88.29% versus 90.50%; HR 1.30 (95% CI, 1.27 to 1.34); log-rank χ2 351.935, P < .001.

COVID-19. Risks were 2.9% versus 1.2%; risk ratio 2.36 (95% CI, 2.26 to 2.46). KM five-year survival 93.72% versus 96.32%; HR 1.70 (95% CI, 1.63 to 1.77); log-rank χ2 618.288, P < .001.

Age 12 to 18 years (post-match n = 232,944 per cohort)

Influenza. Across 5 years, 10,833 of 221,172 (4.9%) with OSA versus 6,286 of 230,221 (2.7%) controls had influenza; risk ratio 1.79 (95% CI, 1.74 to 1.85). KM five-year survival 92.38% versus 94.15%; HR 1.32 (95% CI, 1.28 to 1.37); log-rank χ2 313.962, P < .001.

COVID-19. Risks were 2.3% versus 0.9%; risk ratio 2.52 (95% CI, 2.40 to 2.65). KM five-year survival 96.35% versus 97.96%; HR 1.82 (95% CI, 1.73 to 1.91); log-rank χ2 541.549, P < .001.
